# Different cochleovestibular manifestations and outcomes in patients diagnosed with dengue^☆^

**DOI:** 10.1016/j.bjorl.2017.07.008

**Published:** 2017-09-19

**Authors:** Isabella Marques Pereira Rahme, Geraldo Majela Pereira, Tanit Ganz Sanchez

**Affiliations:** a Universidade Federal de Minas Gerais (UFMG), Belo Horizonte, MG, Brazil; b Otorrino Clínica, Itaúna, MG, Brazil; c Universidade de São Paulo (USP), Faculdade de Medicina, Departamento de Otorrinolaringologia, São Paulo, SP, Brazil; d Instituto Ganz Sanchez, São Paulo, SP, Brazil

## Introduction

Dengue is the most important human arboviral disease and a global public health concern throughout tropical/sub-tropical regions. About 50–100,000,000 people contracted dengue worldwide and 500,000 had the severe form with hospitalization.^1^ The mosquito *Aedes aegypti* transmits four virus serotypes, whose simultaneous movement has determined the hyper endemicity of dengue.

Symptoms include rapid onset of fever, headache, different pains and cutaneous rash. Severe cases (Dengue Hemorrhagic Fever and Dengue Shock Syndrome) have thrombocytopenia, increased edema and hemorrhage due to endothelial cell dysfunction and vascular leak.^2^ Shock occurs after 3–4 days.

Ear, nose and throat symptoms reported in dengue are similar to other viral infections.^2^ However, cochleovestibular symptoms have rarely been reported.

This study describes four patients who presented serologic-confirmed dengue infection and cochleovestibular manifestations and showed heterogeneous outcomes.

## Case reports

### Case 1

**Figure 1:**
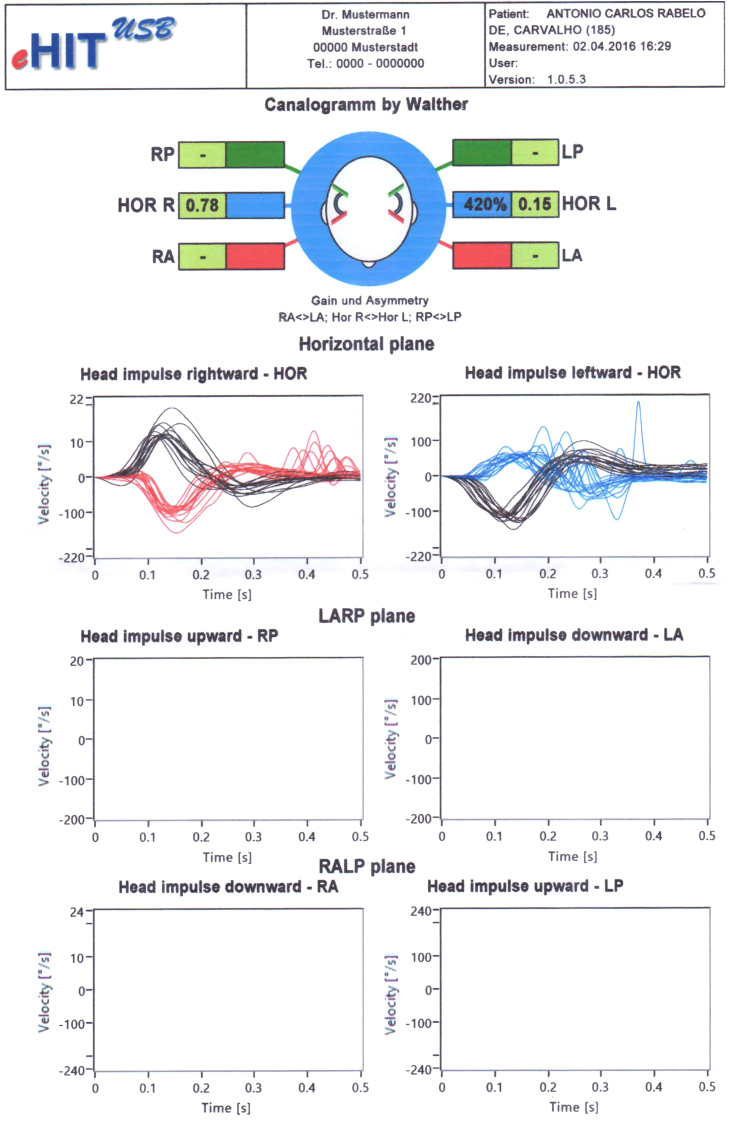
Patient's first vHIT after dengue: saccades at the end of each head rotation (blue traces).

ACRC, male, 56, reported the first episode of intense vertigo five days after beginning dengue infection (April 2016). Physical exam was normal. Video Head Impulse Test (vHIT) was 0.78 (right ear) and 0.16 (left ear), which characterized left unilateral vestibular dysfunction (Fig. 1). vHIT enables the visualization of vestibuloocular reflex and objectifies disorders of semicircular canals. It represents the ratio of the area under the curves of the eye to head movement (set by the device). Results <0.6 are considered abnormal.^3^

**Figure 2:**
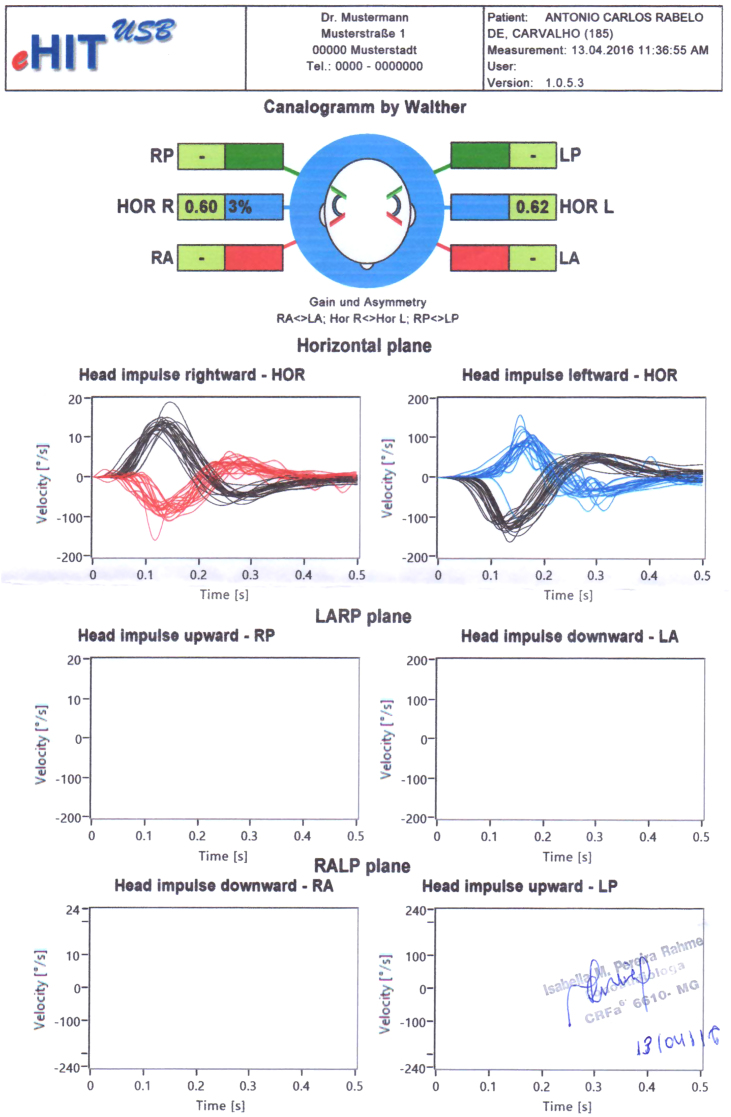
Patient's second vHIT (after treatment) with bilateral normal gain. Figure superimposes records of head velocity stimulus (black traces) and slow-phase eye-velocity responses (blue and red traces) to brief unpredictable head turns in the direction of horizontal semicircular canal. Tiny overt catch-up saccades are normal in healthy subjects.

Patient was prescribed prednisolone plus meclizine for 5 days and vestibular rehabilitation (Cawthorne and Cooksey protocol). After 11 days, he was asymptomatic and vHIT was normal (Fig. 2).

### Case 2

MNFM, female, 63, reported sudden hearing loss, tinnitus and vertigo four days after beginning dengue infection (April 2016). Physical exam was normal. Audiometry showed left profound sensorineural hearing loss. Brain/ear magnetic resonance was normal. After 12 days, tinnitus and vertigo improved spontaneously, but left deafness persisted.

### Case 3

**Figure 3:**
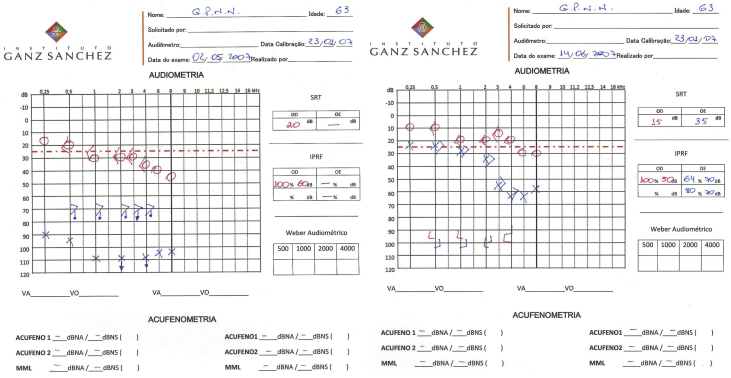
Patient's first audiometry (left) after perceiving sudden hearing loss and tinnitus (May, 2007) and second audiometry (right) after treatment, with partial improvement of hearing loss and tinnitus (June, 2007).

**Figure 4:**
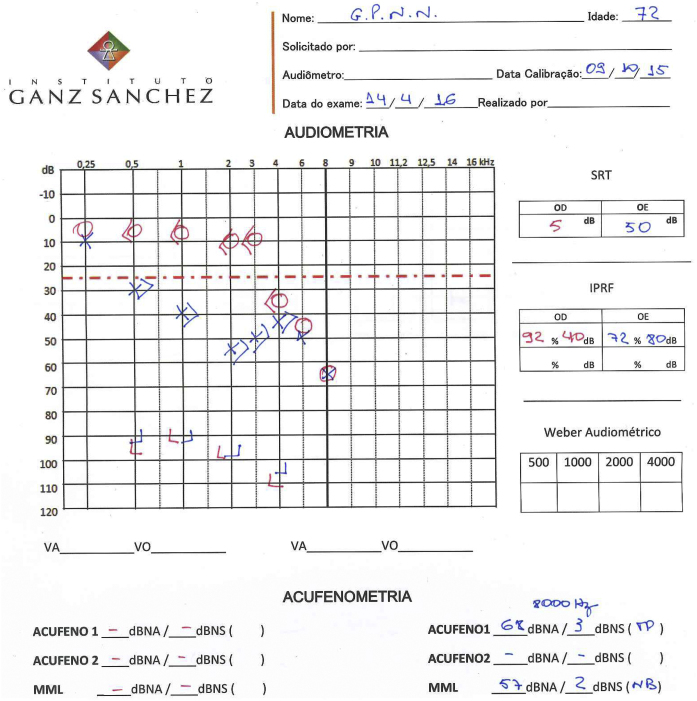
Patient's third audiometry after a nine-year interval (April 2016), presenting stable hearing loss and low-level tinnitus loudness.

GPNN, male, 63, reported sudden hearing loss and high frequency tinnitus in the left ear about one week after beginning the dengue infection (May 2007). Painful vesicle lesions were detected in left pinna and co-infection by herpes zoster oticus virus (HZO or Ramsay-Hunt syndrome) was suspected as a possible complication. No facial paralysis or balance disorders were reported. Audiometry showed left profound hearing loss (Fig. 3). He was prescribed corticosteroid and acyclovir for ten days. After one month, physical exam was normal and he reported partial improvement of hearing loss and tinnitus. The second audiometry showed a moderate sensorineural hearing loss and Speech Recognition Threshold (SRT) was 64% (monosyllables) and 80% (disyllables) in the left ear (Fig. 3). The discomfort with hearing loss and tinnitus through the 0–10 numeric scale decreased from 7 and 8 to 3 and 3. Patient lost follow-up until April 2016, when he was 72. In this 9 year interval, he remained stable. His third audiometry showed stability of hearing loss, with SRT = 72% (monosyllables). Tinnitus pitch and loudness matching showed that tinnitus was 3 dBSL at 8000 Hz (Fig. 4).

### Case 4

MFD, female, 38, had two episodes of dengue. In the first (May 2006), she reported high fever with generalized severe pain. She recovered well. In the second episode (April 2015), pain was more intense, more restricted to wrists and ankles, associated to fever, major fatigue, and increased platelet fall. In addition, she reported bilateral tinnitus and hyperacusis (discomfort with loudness of everyday sounds), whose discomfort was defined as 8 and 6, respectively. When the dengue infection was over, both ear symptoms disappeared. She came to the appointment asymptomatic. The audiogram was bilaterally normal. After 2 years, a follow-up made by telephone indicated that no recurrence occurred.

## Discussion

The highlights of this study are to describe:

1. The presence of variable combinations of cochleovestibular symptoms in patients with serologic-confirmed dengue infection (Table 1): tinnitus (*n* = 3), vertigo (*n* = 2), sudden hearing loss (*n* = 2), sound intolerance (*n* = 1). No patient has ever reported such symptoms before.

**Table 1:** Combination of cochleovestibular symptoms in patients with serologic-confirmed dengue infection and their outcomes.

	Vertigo	Hearing loss	Tinnitus	Hyperacusis	Outcome
ACRC ♀ 56	Present	Absent	Absent	Absent	Complete improvement after treatment
MNFM ♀ 63	Present	Present	Present	Absent	Spontaneous partial improvement
GPNN ♂ 73	Absent	Present	Present	Absent	Partial improvement after treatment
MFD ♀ 38	Absent	Absent	Present	Present	Spontaneous complete improvement
2. The heterogeneous outcomes: vertigo had complete improvement after treatment in ACRC (Case 1); tinnitus and sound intolerance (MFD) (Case 2) had spontaneous disappearance when dengue was over; sudden hearing loss accompanied by tinnitus had partial recovery after treatment (GPNN) (Case 3) but no recovery in MNFM (Case 2), although she had partial improvement of tinnitus and vertigo (Table 1).

Sudden sensorineural hearing loss (SSHL) is associated to viral infection, vascular or immunologic factors. It can present with tinnitus and vertigo,^4^ as in patient MNFM (Case 2), whose tinnitus and vertigo improved spontaneously, but SSHL remained unchanged. Three mechanisms justify the SSHL: 1) viral invasion of the cochlear fluids/cochlear nerve via hematogenous/cerebrospinal fluid routes or middle ear; 2) reactivation of dormant neurotropic virus in cochlear nerve; 3) systemic/distant viral infection triggering immune-mediated response in the inner ear.^2^, ^4^

Specifically in Ramsay Hunt syndrome or Herpes Zoster Oticus (HZO), patients usually present unilateral ear pain, vesicle lesions in pinna and facial weakness/paralysis. Hearing loss, tinnitus and vertigo only occur in 20% and have sudden onset because herpes zoster virus cause intense acute inflammatory reaction in VII/VIII cranial nerves.^5^ Among 81 patients with HZO, facial weakness, hearing loss, and vertigo were the main symptoms. Ipsilateral facial weakness was present in 62/81 cases and the interaction between vertigo and hearing loss is interesting: almost all patients with vertigo also had hearing loss (28/30), and patients without hearing loss did not have vertigo (19/21).^6^ Patient GPNN presented typical vesicle lesions in the pinna, SSHL and tinnitus, with no vertigo/facial paralysis. Partial improvement of hearing loss and tinnitus remained stable for 9 years.

Patient ACRC (Case 1) developed intense vertigo during dengue. The main hypothesis was vestibular neuritis, which involves reactivation of latent infection of vestibular ganglion by herpes simplex virus Type I, leading to unilateral vestibular dysfunction. Symptoms include oscillopsia and nausea, horizontally rotating spontaneous nystagmus to the non-affected side, gait deviation and tendency to fall to the affected side, which persist for many days.^3^ The vHIT shows impaired function of vestibulo-ocular reflex when patient turns to the affected ear. Patient's first result characterized left vestibular dysfunction. We wonder whether vestibular neuritis is associated with dengue virus or reactivation of latent herpes simplex virus Type I or immune-mediated reaction may be involved.^2^, ^4^ Patient ACRC (Case 1) was asymptomatic after treatment, while patient MNFM (Case 2) presented vertigo that spontaneously disappeared after 12 days before the medical consultation.

Regarding tinnitus, the sound perception in the absence of external sources, it reduces quality of life for millions worldwide.^7^ It is a multifactorial condition and association with hearing loss in the audiogram is evident. Moreover, 25–40% of patient's also present hypersensitivity to sounds (hyperacusis). Both symptoms could reflect synaptic damage to high-threshold auditory nerve fibers that is not detected by the audiogram (“hidden hearing loss”).^8^ In animal studies, such damage is induced by noise trauma^9^ and further induces compensatory changes in central auditory pathways that increase behavioral responses to sound stimuli.^10^ However, no report of hidden hearing loss was found in association with dengue virus. Three patients started tinnitus during dengue and have partial or total improvement: MNFM (Case 2) and GPNN (Case 3), who also had hearing loss, and MFD (Case 4), who also had hyperacusis.

All cases had previously performed serologic confirmation of dengue. It can be performed by: a) Molecular testing, which detects the dengue virus in blood within the first week after fever appears (the positive result is very conclusive); b) Antibody tests: the presence of IgM antibodies means that the person was recently infected by dengue virus. It can have cross-reaction with chikungunya virus. Then, a second test (Plaque Reduction Neutralization Test) confirms the presence of antibodies to dengue virus and rules out other viral infections.

As cochleovestibular symptoms are not often described during dengue, we wonder whether they might be present in other arboviruses like zika, chikungunya and yellow fever.

## Conclusion

Patients with serologic-confirmed dengue infection may present concomitant cochleovestibular symptoms that were manifested for the first time. Their outcomes were heterogeneous. As cochleovestibular manifestations are poorly described in dengue literature, we call attention of health professionals to possible higher prevalence than estimated in this and other arboviral diseases.

## Conflicts of interest

The authors declare no conflicts of interest.
